# Aspalathin, a natural product with the potential to reverse hepatic insulin resistance by improving energy metabolism and mitochondrial respiration

**DOI:** 10.1371/journal.pone.0216172

**Published:** 2019-05-02

**Authors:** Sithandiwe E. Mazibuko-Mbeje, Phiwayinkosi V. Dludla, Rabia Johnson, Elizabeth Joubert, Johan Louw, Khanyisani Ziqubu, Luca Tiano, Sonia Silvestri, Patrick Orlando, Andy R. Opoku, Christo J. F. Muller

**Affiliations:** 1 Biomedical Research and Innovation Platform, South African Medical Research Council, Tygerberg, South Africa; 2 Division of Medical Physiology, Faculty of Health Sciences, Stellenbosch University, Tygerberg, South Africa; 3 Department of Life and Environmental Sciences, Polytechnic University of Marche, Ancona, Italy; 4 Plant Bioactives Group, Post-Harvest and Agro-Processing Technologies, Agricultural Research Council, Infruitec-Nietvoorbij, Stellenbosch, South Africa; 5 Department of Food Science, Stellenbosch University, Stellenbosch, South Africa; 6 Department of Biochemistry and Microbiology, University of Zululand, KwaDlangezwa, South Africa; Max Delbruck Centrum fur Molekulare Medizin Berlin Buch, GERMANY

## Abstract

Aspalathin is a rooibos flavonoid with established blood glucose lowering properties, however, its efficacy to moderate complications associated with hepatic insulin resistance is unknown. To study such effects, C3A liver cells exposed to palmitate were used as a model of hepatic insulin resistance. These hepatocytes displayed impaired substrate metabolism, including reduced glucose transport and free fatty acid uptake. These defects included impaired insulin signaling, evident through reduced phosphatidylinositol-4,5-bisphosphate 3-kinase/ protein kinase B (PI3K/AKT) protein expression, and mitochondrial dysfunction, depicted by a lower mitochondrial respiration rate. Aspalathin was able to ameliorate these defects by correcting altered substrate metabolism, improving insulin signaling and mitochondrial bioenergetics. Activation of 5ʹ-adenosine monophosphate-activated protein kinase (AMPK) may be a plausible mechanism by which aspalathin increases hepatic energy expenditure. Overall, these results encourage further studies assessing the potential use of aspalathin as a nutraceutical to improve hepatocellular energy expenditure, and reverse metabolic disease-associated complications.

## Introduction

Obesity and dysglycemia, the major characteristic features of the metabolic syndrome, have become prevalent in the general population and are associated with a rapid rise in morbidity and mortality [1, 2]. Insulin resistance, mostly driven by an imbalance between intake and utilization of metabolic substrates such as carbohydrates and lipids remains a prominent hallmark of the metabolic syndrome [3]. Since described by Randle in 1963 as an important biochemical mechanism in the development of insulin resistance [4], increasingly research has focused on effective regulation of glucose and free fatty acid (FFA) metabolism to control diabetes and its associated complications [5–9]. Based on these studies, it is now well-accepted that impaired glucose and FFA metabolism affects optimal functioning of major organs in the human body, including adipose tissue, skeletal muscle, myocardium and liver. In fact, abnormal substrate metabolism has been correlated with reduced insulin sensitivity and glucose transport in the liver [10]. The liver plays an essential role in the physiological regulation of whole-body energy homeostasis and in the pathogenesis of the epidemiologically relevant metabolic disorders [11]. Diets rich in saturated fat and sugar content that are increasingly consumed in industrialized and developing societies, together with lack of physical activity contribute greatly to the development of pathological conditions, such as obesity, hypertension, insulin resistance, and liver diseases [12]. Through various experimental models, for example, high fat diet-fed rodents or palmitate exposed hepatocytes, abnormal FFA metabolism has also been linked with defective insulin signaling, mainly through the impairment of the phosphatidylinositol-4,5-bisphosphate 3-kinase/ protein kinase B (PI3K/AKT) pathway [10, 13, 14].

Despite the acknowledged importance of the PI3K/AKT pathway in insulin signaling and cell survival [15], complete mechanisms underlying insulin resistance in the liver are not fully understood. For instance, 5ʹ-adenosine monophosphate-activated protein kinase (AMPK) appears to be another instrumental process in controlling cellular energy metabolism, including some mechanisms involved in the development of insulin resistance [16, 17]. In the liver, AMPK may modulate FFA metabolism through the control of mitochondrial function, effects which can be mediated through coordinated regulation of mitochondrial bioenergetics. As recently reviewed [18], altered AMPK expression is linked with the development of nonalcoholic fatty liver disease (NAFLD), a clinical feature that is associated with obesity, insulin resistance, and type 2 diabetes. Consistently, reviewed models also show that liver-specific inhibition of AMPK is usually followed by reduced mitochondrial content and mitochondrial respiration indicating that AMPK is important for hepatic mitochondrial function [18, 19]. Thus, it remains of interest to develop novel drugs that act broadly to control energy metabolism, improve mitochondrial function and increase hepatocellular energy expenditure, potentially leading to the reversal of metabolic complications.

**Fig 1:**
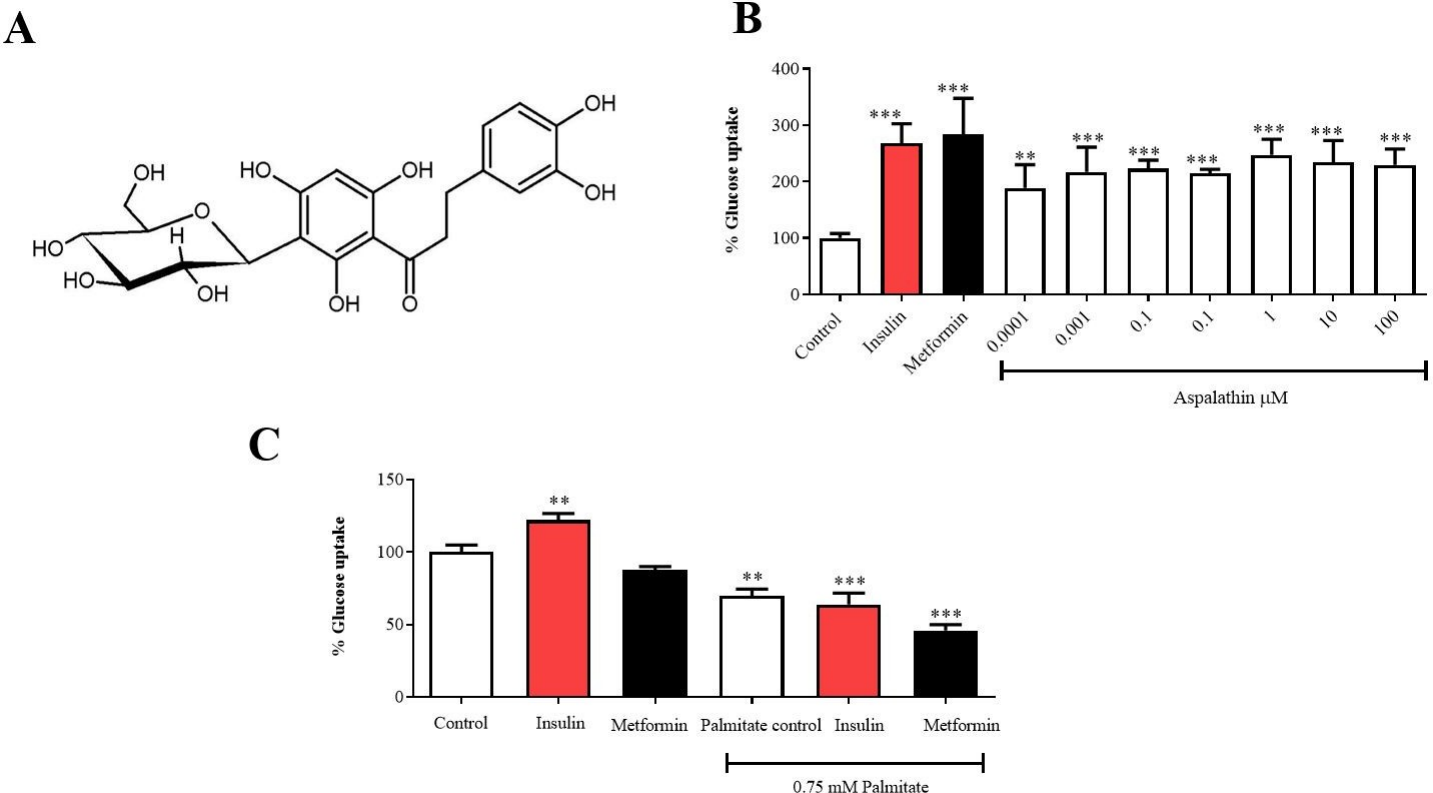
Chemical structure of aspalathin (**A**), dose dependent effect of aspalathin on improving glucose uptake (**B**), and the effect of insulin and metformin in palmitate-exposed C3A cells (**C**). Results are expressed as mean ± SEM of three independent experiments. ** p < 0.01, *** p < 0.001 versus normal control.

Nutraceuticals have garnered a lot of interest for their therapeutic potential in delaying aging and reducing chronic diseases, with an effort to increase life expectancy [20]. Our group is exploring the metabolic benefits and potential use of bioactive compounds present in *Aspalathus linearis*, a South African plant used to manufacture the widely consumed rooibos herbal tea, as nutraceuticals [21–23]. Of particular interest, is aspalathin (Fig 1A), a *C*-glucosyl dihydrochalcone with blood glucose and lipid lowering properties [24–27]. This flavonoid can modulate both PI3K/AKT and AMPK pathways to improve substrate metabolism in the adipose tissue, and skeletal and cardiac muscle [8, 27, 28]. Although an aspalathin-enriched green rooibos extract has displayed moderate effect in improving liver function in an insulin-resistant state [29], currently no study has reported on the impact of aspalathin on the metabolic syndrome associated hepatic complications. Using palmitate-exposed liver cells as a model of insulin resistance, we report on the ameliorative effect of aspalathin against altered substrate metabolism. We further demonstrate that treatment of cells with aspalathin was associated with improved mitochondrial respiration and enhanced energy expenditure under conditions of insulin resistance.

## Materials and methods

### Chemicals and reagents

Aspalathin (ca 98%, Batch SZI-356-54), synthesized following an already published method by Han et al. [30], was supplied by High Force Research LTD (Durham, UK). Human C3A liver cells (ATTC Cat. No. CRL-10741) were from the American Type Culture Collection (Manassas, VA, USA). 2-Deoxy-[^3^H]-D-glucose and ^14^C palmitate were obtained from American Radiolabeled Chemicals (St Louis, MO, USA), while Bradford and RC DC protein assay kits, and Mini-PROTEAN TGX Stain-Free Precast Gels, nitrocellulose and PVDF membranes were from Bio-Rad Laboratories (Hercules, CA, USA). Eagle's Minimum Essential Medium (EMEM), Dulbecco’s phosphate buffered saline (DPBS, pH 7.4 with calcium and magnesium), penicillin-streptomycin and MycoAlert mycoplasma detection and ViaLight plus ATP kits were from Lonza (Basel, Switzerland), while fetal calf serum (FCS) was obtained from Biochrom (Berlin, Germany). Chemiluminescence (ECL) kit was from Amersham Bioscience (Westborough, MA, USA). Primary antibodies raised against AKT (cat# 9272S), p-AKT (Ser473) (cat# 9271S), AMPK (cat# 2532), p-AMPK (Thr172) (cat# 2535S), carnitine palmitoyltransferase 1A (CPT1) (D3B3) (cat# 12252S), PI3K (cat# 4257S), p-PI3K (p85) (cat# 4228S) were from Cell Signalling Technology (Beverly, MA, USA), while glucose transporter (GLUT) 2 (cat# ab54460) was from Abcam (Cambridge, UK). The reference control, β-actin, and secondary antibodies, goatanti-mouse and goatanti-rabbit IgG–horseradish peroxidase, were purchased from Santa Cruz Biotechnology (Dallas, TX, USA). Seahorse XF96 microplate plates, Seahorse XF Assay media, Seahorse XF base media without phenol red and Seahorse XF-Cell Mito Stress, Glycolysis stress assay kits were from Agilent (Santa Clare, CA, USA). All other cell culture reagents were obtained from Sigma-Aldrich (St Louis, MO, USA)

### Cell culture and treatment conditions

Hepatocytes (C3A liver cells; Cat. No. CRL-10741) were obtained from the American Type Culture Collection (Manassas, VA, USA) and cultured in EMEM containing essential amino acids, sodium pyruvate and L-glutamine. Briefly, cell seeding was done on 24-well plates (55 000 cells/well) for 2-deoxy-[^3^H]-D-glucose and ^14^C palmitate uptake experiments, 96-well microplates (11 000 cells/well) for ATP and MTT assays and 6-well plates (165 000 cells/well) for protein analysis. For Seahorse experiments, cells were seeded at 20 000 cells/ well in 96-well Seahorse microplates until confluent. C3A cells were confirmed to be free from mycoplasma before successive experiments, using the MycoAlert mycoplasma detection kit according to the manufacturer’s instructions. Cells were maintained by rinsing with DPBS and trypsinization by adding 2 ml trypsin/ versen to the cell culture, and incubation for 3–7 min until cells dislodged. Thereafter, cells were seeded in EMEM with essential amino acid and L-glutamine for 5-days and incubated under standard culture conditions at (37°C in 5% CO_2_ and humidified air), and media was changed every 48 hours. Cell passages between 5 and 20 were used for all experiments. Insulin resistance was induced by exposing C3A liver cells to 0.75 mM palmitate dissolved in EMEM containing 5.5. mM glucose and 2% bovine serum albumin for 16 h, as previously described [31]. Normal controls were included by culturing in EMEM containing 5.5. mM glucose with 2% bovine serum albumin, but without palmitate. Aspalathin was prepared in dimethyl sulfoxide as previously described [8], and was used at a final concentration of 10 μM in MEM. Since it can interfere with cell viability in some cultured cells [32], the final dimethyl sulfoxide concentrations in the media for aspalathin was < 0.001%. The glucose uptake assay was used to establish optimal concentrations for aspalathin, while insulin and metformin, both at a dose of 1 μM, were employed for 3 h as controls and to confirm the palmitate model, respectively. After the optimal concentration of aspalathin was established, palmitate-exposed C3A liver cells were serum and glucose starved for 30 min before treatment with aspalathin (10 μM) and insulin (1 μM) in EMEM without phenol red (to avoid interference of phenol red with colorimetric assays) for 3 h under standard tissue culture conditions (at 37°C in 5% CO_2_ and humidified air). Briefly, after 16 h media containing palmitate was aspirated and DPBS was added for 30 min to cells as it contains no calcium magnesium and no glucose, so it removes all the chelators from the culture before new treatments are added.

### 2-Deoxy-[^3^H]-D-glucose and ^14^C palmitate uptake assays

2-Deoxy-[^3^H]-D-glucose and ^14^C palmitate uptake assays were performed separately, as previously described [8, 26]. Briefly, after 3-h culture with relevant treatments, cells were exposed for 3 h under standard tissue culture conditions to 0.5 μCi/mL 2-deoxy-[^3^H]-D-glucose or 1 μCi/mL ^14^C palmitate in medium containing aspalathin. Insulin (1 μM), the positive control, was added during the last 15 min in each experiment. Thereafter, cells were lysed with 0.1 M NaOH before 2-deoxy-[^3^H]-D-glucose and ^14^C palmitate activity in the lysate was assessed by liquid scintillation (2220 CA, Packard Tri-Carb series, PerkinElmer, Downers Crove, IL, USA).

### Metabolic activity assays

Intracellular ATP was measured using the ViaLight plus ATP kit, following manufacturer’s recommendations, whereas the MTT (3-(4,5-dimethylthiazol-2-yl)-2,5-diphenyltetrazolium bromide) assay was done according to a previously described method [33]. For the ATP assay, luminescence was measured using a BioTek FLX 800 plate reader (BioTek Instruments Inc., Winooski, VT, USA), while absorbance as indicator of MTT reduction was detected at 570 nm, using BioTek ELX 800 plate reader. Gen 5 software was used for data acquisition.

### Cellular respiration measurements

Oxygen consumption rate (OCR) and extracellular acidification rates (ECAR) were measured with the Mito Stress assay kit, using the XF96 Extracellular Flux analyzer from Agilent. Briefly, C3A liver cells were plated into cell culture XF96 microplate plates at 20,000/well for 48 h before treated as previously described in the cell culture and treatment section. Following treatment, cells were washed twice with 180 μL of pre-warmed assay medium (XF base medium supplemented with 10 mM glucose, 2 mM glutamine and 1 mM sodium pyruvate; pH 7.4), thereafter, cells were incubated in 180 μL XF Assay Medium at 37°C without CO_2_ for 60-min to equilibrate temperature and pH prior to OCR. The XF96 plate was then transferred to a temperature-controlled (37°C) Seahorse XF96 extracellular flux analyzer where it was subjected to a 10 min equilibration period at three assay cycles, each including a 1 min mix, 3 min wait and 3 min measure period cycle. Following this, oligomycin (1 μM) was injected in port A to inhibit ATP synthase. After 3 assay cycles, 75 μM carbonyl cyanide 4 trifluoromethoxy-phenylhydrazone (FCCP) was added in port B to measure maximal respiration. A mixture of Complex I inhibitor (rotenone) and complex III inhibitor (antimycin) was injected in port C to shut down mitochondrial respiration and enable the calculation of non-mitochondrial respiration. At the end of the incubation period, the plates were used to assess protein concentration in each well by Bradford protein assay. OCR (pmol/min) was normalized based on protein content. OCR and ECAR were reported as absolute rates (pmoles/min for OCR and mpH/min for ECAR).

### Glycolysis stress test

After a 3 h incubation with relevant treatment, the medium was changed to assay medium (XF base medium DMEM without phenol red supplemented with 2 mM glutamine), and cells were incubated in a non-CO_2_ incubator at 37°C for 60 min before the assay. For measurement of acute glycolysis, the compound of interest (aspalathin) or buffer was injected in port A followed by injections of glucose (10 mM), oligomycin (1 μM) and 2-DG (100 mM) into ports B, C and D, respectively. The instrument was calibrated, and the assay was performed using a glycolytic stress test assay protocol as recommended by the manufacturer (Agilent Technologies). ECAR was measured under basal conditions followed by the sequential addition of aspalathin, 10 mM glucose, 0.5 μM oligomycin, and 100 mM 2-DG (ASP> Glu> Oli> 2-DG). Thereafter non-glycolytic acidification, glycolysis, glycolytic capacity and glycolytic reserve were calculated using Wave desktop (Agilent Technologies).

### Western blot analysis

A standardized protocol was used for Western blot analysis [34]. Briefly, Mini-PROTEAN TGX stain-free precast or 10% SDS-PAGE gels were used to separate denatured proteins (20 or 40 μg) before being transferred to PVDF-P or nitrocellulose membranes. Low-fat milk powder in Tris-buffered saline (w/v) containing Tween 20 was used to block non-specific protein labeling. Thereafter, membranes were labeled overnight at 4°C with relevant primary antibodies (AKT, p-AKT (Ser473), AMPK, GLUT2, p-AMPK (Thr172), CPT1 and PI3K, p-PI3K (p85)). The following day, horseradish peroxidase-conjugated secondary antibody was applied for 1.5 h. Proteins were detected and quantified by chemiluminescence using a Chemidoc-XRS imager and Quantity One 1-D software, while molecular weight band detection was confirmed using ImageJ software (Bio-Rad Laboratories), respectively. β-actin was used as the reference control. Relevant material for Western blot analysis, including sources for antibodies used for experimental work and images used for data quantification, is included as part of the supporting information (S1–S6 Datasets and S1 Table).

### Statistical analysis

Data are presented as the mean ± SEM of three independent experiments. GraphPad Prism 5 software (GraphPad Software Inc. La Jolla, CA, USA) was used for all statistical analyses. Comparisons between treatment groups were performed by one-way analysis of variance (ANOVA), followed by a Tukey post hoc test and Student *t*-test. A p value of < 0.05 was considered statistically significant. For cellular respiration experiments, the XF Mito Stress test report generator and the XF glycolysis stress test report generator automatically calculated the respective parameters from Wave data that have been exported to Excel. Respiration and acidification rates are presented as the mean ± SEM of 2 independent experiments in all experiments performed, with 4 to 8 replicate wells in the Seahorse XF96 analyzer. For the experiment of the effect of energy substrates on mitochondrial respiration, the significance level was determined by performing ANOVA on the complete data set with Tukey׳s post-hoc testing. The results were considered significant at p < 0.05.

## Results

### Establishing the effective dose of aspalathin

Glucose uptake assay was used to determine the most active concentration of aspalathin in C3A liver cells. Results showed that together with both insulin and metformin, all tested concentrations of aspalathin (0.0001–100 μM) improved glucose uptake in cells (Fig 1B). However, a dose of 10 μM was deemed most effective and selected for subsequent experiments (Fig 1C). Furthermore, an insulin resistance model was established by exposing cells with palmitate before treatment with well-known antidiabetic drugs, insulin and metformin (Fig 1C). This model was successfully established, as demonstrated by a significant reduction (p < 0.005) in glucose uptake when compared to cells in the control group (Fig 1C).

### Aspalathin improved substrate metabolism in insulin-resistant hepatocytes

**Fig 2:**
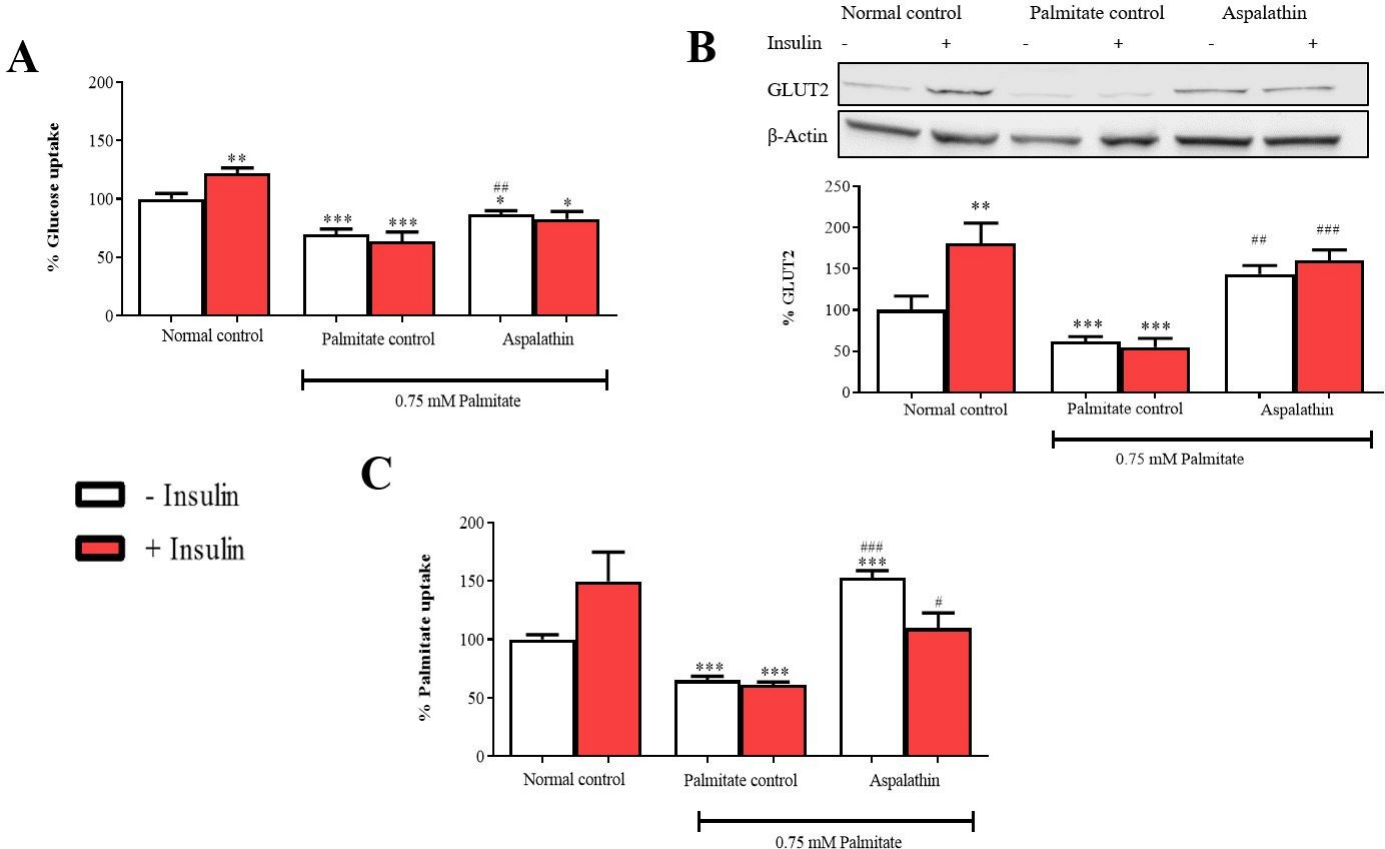
The effect of aspalathin on glucose uptake (**A**), glucose 2 protein expression (**B**), and palmitate uptake (**C**) in insulin resistant C3A liver cells. Results are expressed as mean ± SEM of three independent experiments. * p < 0.05, ** p < 0.01, *** p < 0.001 versus normal control (no insulin); ^#^ p < 0.05, ^##^ p < 0.01, ^###^ p < 0.001 versus palmitate control (no insulin).

Like in many cells, FFAs and glucose are the major substrates utilized by hepatocytes to generate energy. Impaired hepatic substrate metabolism was confirmed by a significant reduction in FFA and glucose uptake after palmitate exposure (p < 0.001) (Fig 2A, 2B and 2C). A similar effect was observed with protein expression of GLUT2 (p < 0.001), the main transporter of glucose in hepatocytes (Fig 2C). Treatment with aspalathin effectively ameliorated these effects resulting in increased FFA (p < 0.001), and glucose (p < 0.01) uptake and GLUT2 protein expression (p < 0.01) (Fig 2A, 2B and 2C). Apart from enhancing GLUT2 expression, insulin stimulation did not provide a major improvement in FFA and glucose uptake.

### Aspalathin moderately improved insulin signaling by regulating PI3K/AKT pathway in palmitate-exposed hepatocytes

**Fig 3:**
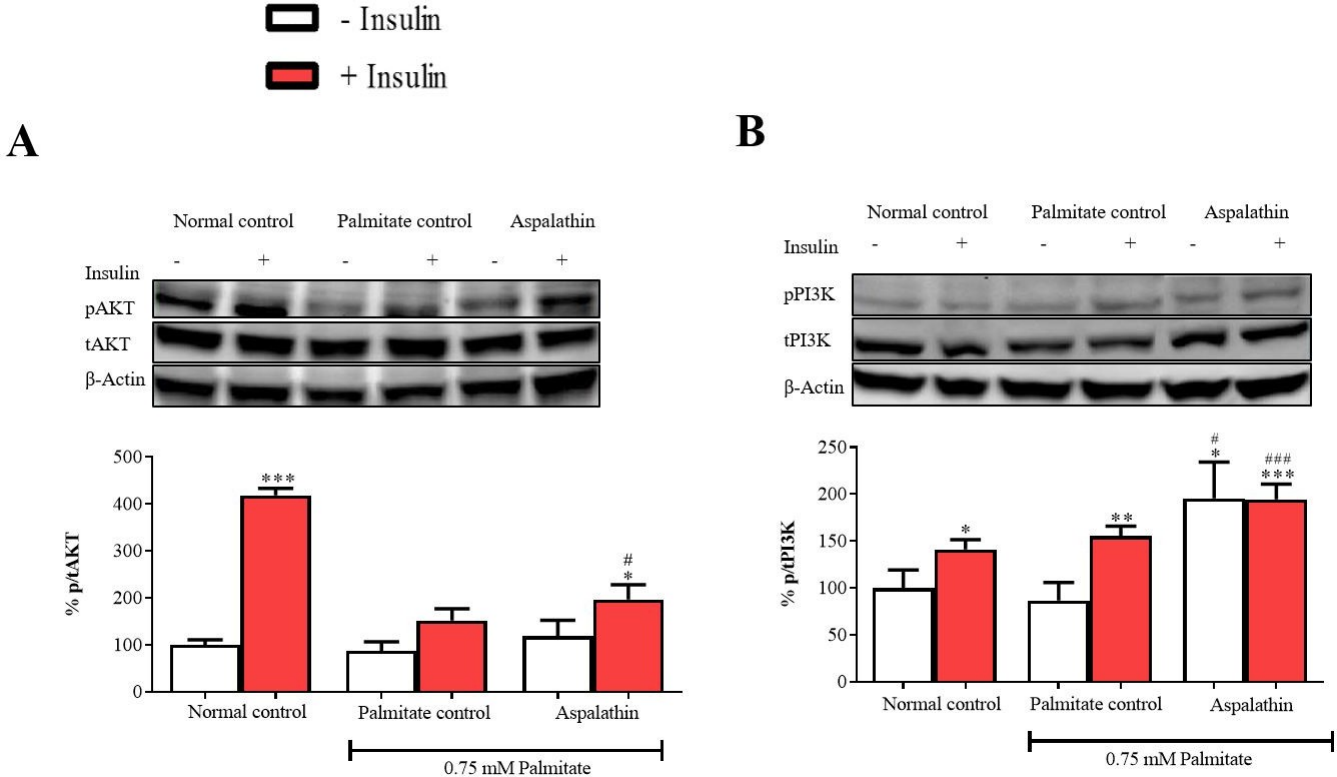
The effect of aspalathin on protein kinase B (AKT) protein expression (**A**), and phosphatidylinositol-4,5-bisphosphate 3-kinase (PI3K) protein expression (**B**) in insulin resistant C3A liver cells. Results are expressed as mean ± SEM of three independent experiments. * p < 0.05, ** p < 0.01, *** p < 0.001 versus normal control (no insulin); ^#^ p < 0.05, ^###^ p < 0.001 versus palmitate control (no insulin).

The PI3K/AKT pathway represents one of the most investigated mechanisms in connection with pathological complications of insulin resistance. Although no changes were observed under basal conditions, exposure of hepatocytes to elevated palmitate concentrations reduced phosphorylation of AKT (p < 0.001) in insulin-stimulated cells when compared to the normal control (Fig 3A). No significant differences were observed for PI3K phosphorylation after exposure to palmitate, however, marked difference was seen under insulin-stimulated conditions (Fig 3B). While treatment with aspalathin improved phosphorylation of PI3K (p < 0.001), that of AKT was only observed under conditions of insulin stimulation (p < 0.05), in comparison to the insulin resistant group (Fig 3A and 3B).

### Aspalathin activated AMPK to regulated FFA oxidation in palmitate-exposed hepatocytes

**Fig 4:**
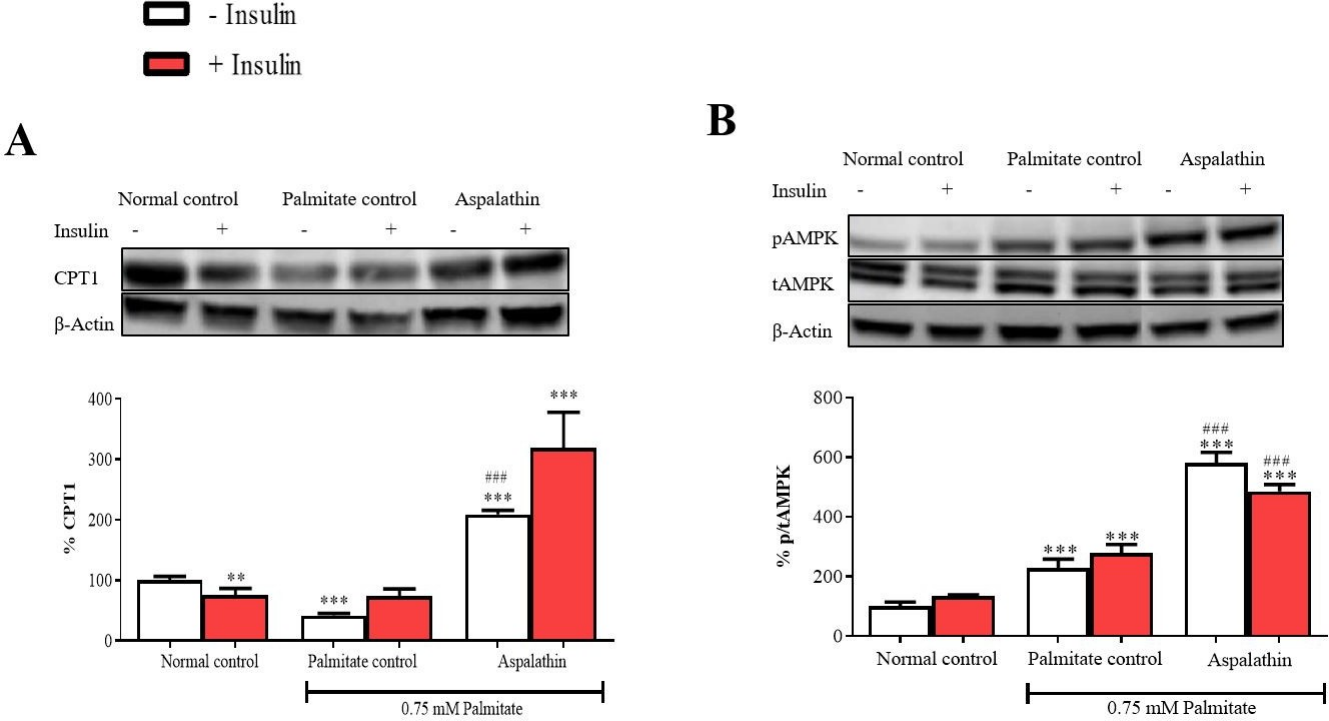
The effect of aspalathin on carnitine palmitoyltransferase 1 (CPT1) protein expression (**A**), and AMP-activated protein kinase (AMPK) protein expression (**B**) in palmitate exposed C3A cells. Results are expressed as mean ± SEM of three independent experiments. * p < 0.05, ** p < 0.01, *** p < 0.001 versus normal control (no insulin); ^#^ p < 0.05, ^##^ p < 0.01, ^###^ p < 0.001 versus palmitate control (no insulin).

Modulation of AMPK, together with CPT1 is increasingly targeted for the regulation of energy metabolism, especially FFA oxidation. Exposure of hepatocytes to elevated palmitate concentrations significantly reduced protein expression of CPT1 (p < 0.001), and slightly enhanced AMPK (p < 0.001) (Fig 4A and 4B). However, treatment of cells with aspalathin markedly upregulated the expression of both proteins (both p < 0.001). Insulin stimulation only had an effect in further enhancing CPT1 expression with aspalathin treatment (Fig 4A).

### Aspalathin enhanced metabolic activity in palmitate-exposed hepatocytes

**Fig 5:**
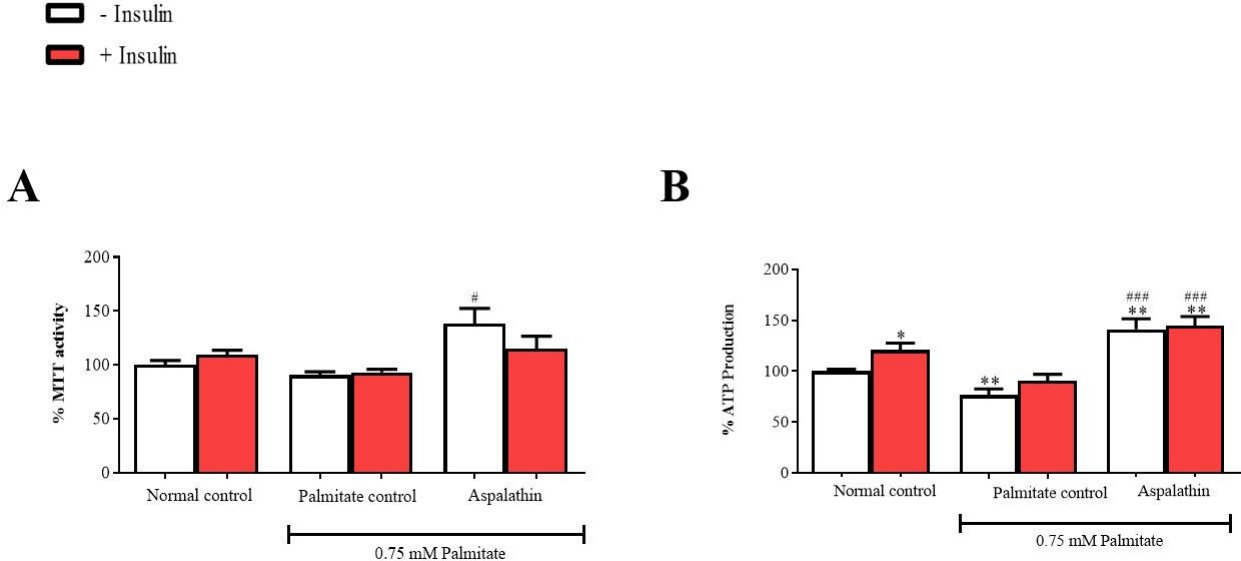
The effect of aspalathin on metabolic activity in insulin-resistant C3A liver cells, as measured by assessing MTT activity (**A**) and ATP production (**B**), respectively. Results are expressed as mean ± SEM of three independent experiments. * p < 0.05, ** p < 0.01 versus vehicle normal control (no insulin); ^#^ p < 0.05, ^###^ p < 0.001 versus palmitate control (no insulin).

The MTT and ATP monitoring assays are widely used to measure the metabolic activity of viable cells. While no effect was observed after cells were exposed to high palmitate concentrations with MTT assay, ATP production was significantly reduced (p < 0.01) when compared to the control (Fig 5A and 5B). However, aspalathin treatment improved metabolic activity of cells. Furthermore, it was evident that insulin stimulation did not affect metabolic activity.

### Aspalathin improves mitochondrial respiration in palmitate-exposed hepatocytes

**Fig 6:**
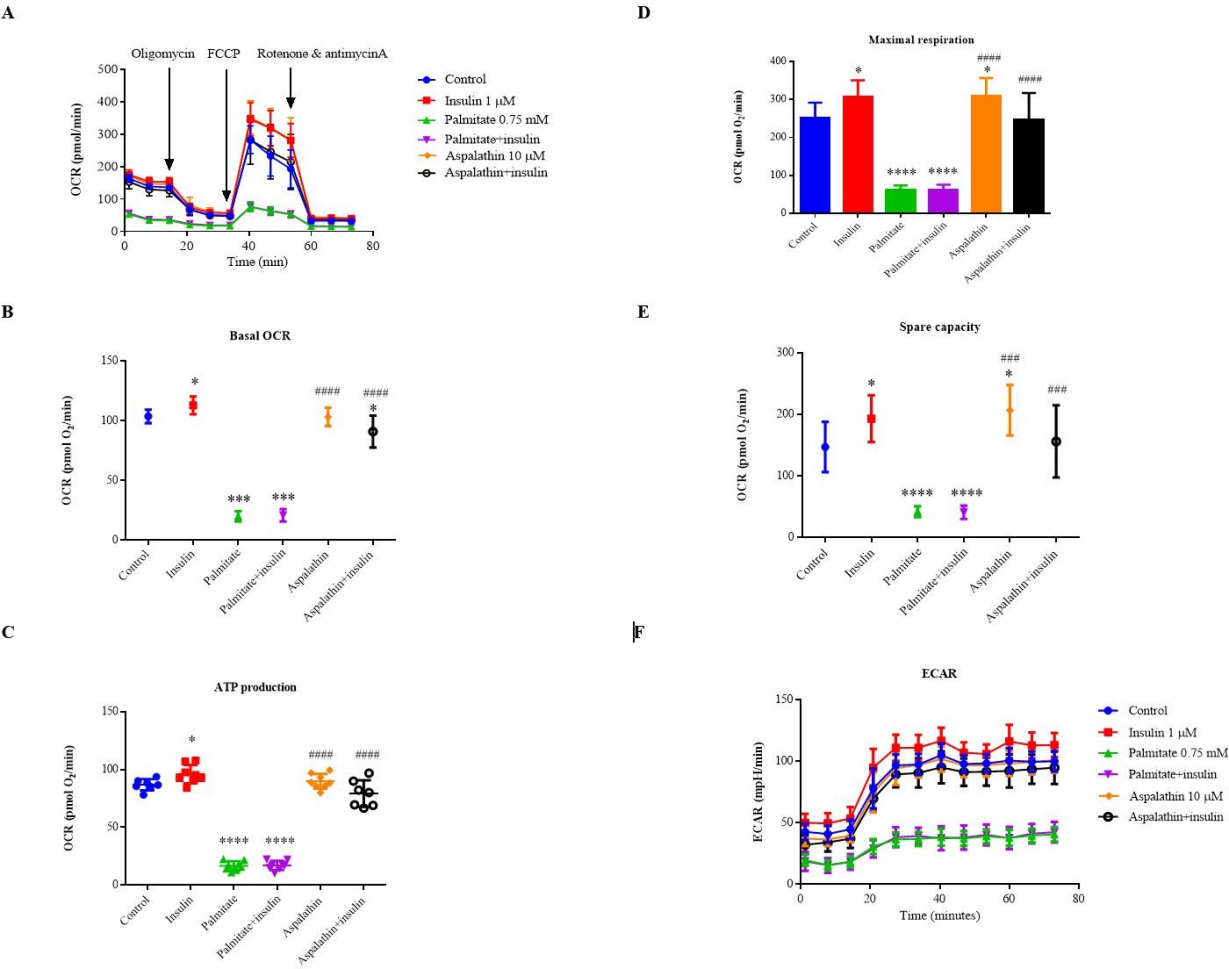
The effects of aspalathin on mitochondrial respiration in insulin-resistant C3A liver cells. The graph shows oxygen consumption rate of all treatments (OCR) (**A**), individual parameters for basal respiration (**B**), ATP production (**C**), maximal respiration (**D**), and spare respiratory capacity (**E**), and extracellular acidification rates (**F**). Oxygen consumption rate was measured under basal conditions followed by the sequential injection of oligomycin (1 μM), FCCP (0.75 μM), as well as rotenone (0.5 μM) & antimycin A (0.5 μM), as indicated. Each data point represents an OCR measurement. Data are expressed as means ± SEM, n = 2 independent experiments. **** p < 0.0001 versus normal (no insulin) control, ^####^ p < 0.0001 versus palmitate control.

Mitochondrial respiration was measured through chronological injections of oligomycin, FCCP, antimycin A and rotenone to assess basal respiration, ATP production, proton leak, maximal respiration, spare respiratory capacity, and non-mitochondrial respiration (Fig 6A–6F). Combined results of OCR after treatment with or without palmitate and aspalathin are shown in Fig 6A and 6B, with individual parameters depicted in Fig 6C–6F. In terms of basal OCR, palmitate significantly reduced both basal and insulin-stimulated OCR (p < 0.0001) when compared to control cells, confirming the high degree of insulin resistance in these cells (Fig 6A and 6B). In agreement with our metabolic assay (Fig 5B), palmitate reduced ATP production (p < 0.0001) (Fig 6D), in maximal respiration (Fig 6E) and spare respiratory capacity (Fig 6F). However, aspalathin treatment significantly reversed these effects, thereby enhancing ATP production and basal, maximal and spare capacity respiration (Fig 6C–6F). The observed effect of aspalathin was comparable to that of the positive control (insulin). In terms of ECAR, palmitate treatment reduced glycolysis, but this reduction was ameliorated by aspalathin treatment with or without insulin (Fig 6F).

### Aspalathin improves glycolytic rate in palmitate-exposed hepatocytes

**Fig 7:**
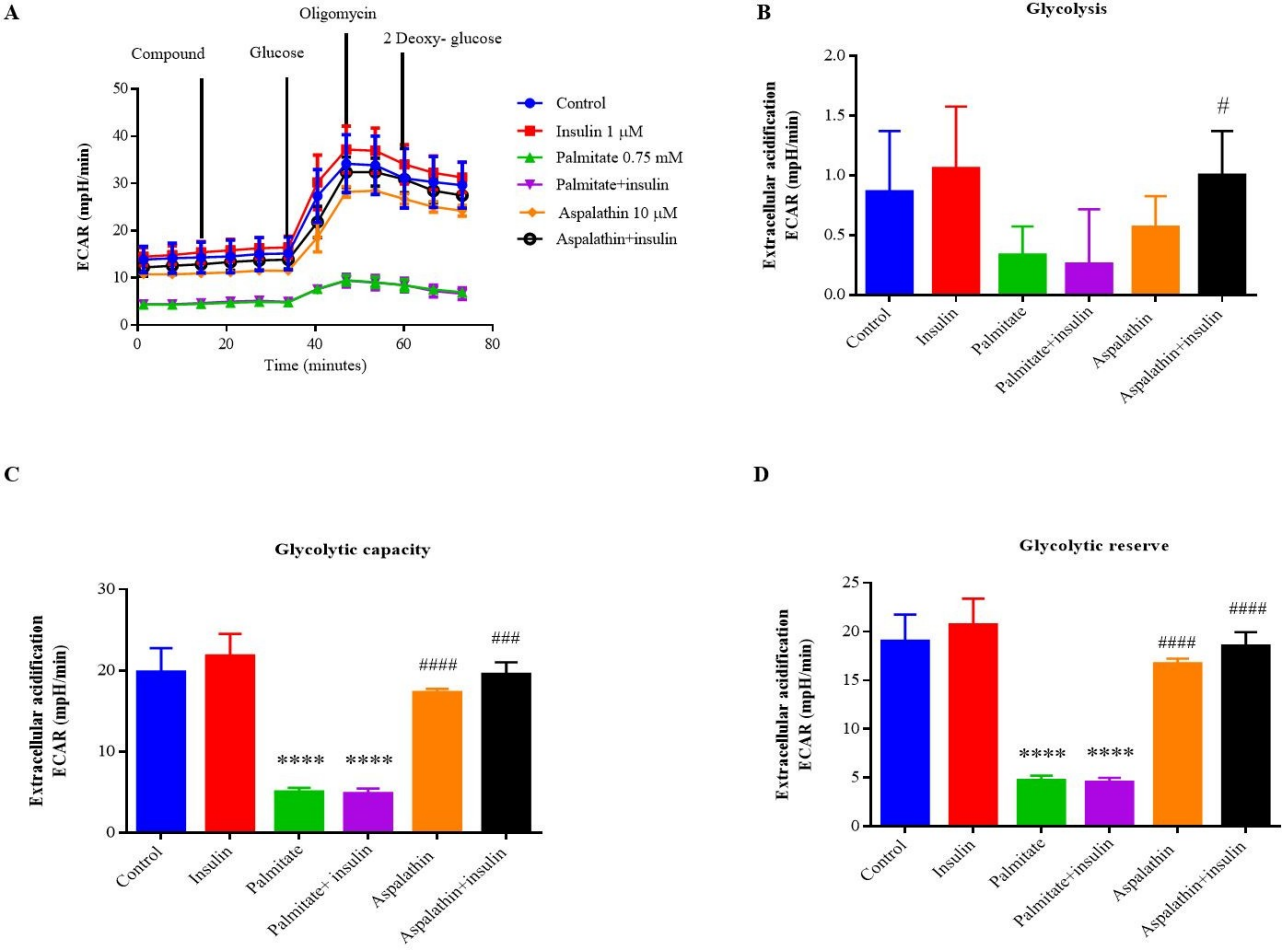
Extracellular acidification rates (ECAR) response of C3A liver cells to aspalathin (10 μM), glucose (10 mM), oligomycin (1 μM) and 2-deoxyglucose (2-DG) (100 mM), respectively (**A**). Individual parameters for glycolysis, glycolytic capacity, glycolytic reserve of C3A liver cells after aspalathin treatment (**B and C**), and the effect of palmitate and aspalathin on OCAR/ECAR (**D**). ECAR was measured under basal conditions followed by the sequential addition of aspalathin (10 μM), glucose (10 mM), oligomycin (1 μM), and 2-deoxyglucose (2-DG) (100 mM) the order was as follows (aspalathin>Glu > Oli > 2-DG). Data are expressed as means ± SEM, n = 3 independent experiments. **** p < 0.0001 versus normal (no insulin) control, ^####^ p < 0.0001 versus palmitate control.

The rate of glucose conversion into pyruvate (glycolysis) was determined after injecting with or without aspalathin in port A, (Fig 7B). Albeit not significant, palmitate reduced glycolysis rate and this slight reduction was reversed to that of the normal control in the aspalathin treated group (Fig 7C). However, conclusions cannot be drawn from this effect, since the effect was not significant. Glycolytic capacity, the measurement for maximum rate of conversion of glucose to lactate by cells showed that palmitate treatment reduced glycolytic capacity (p < 0.001). This reduction was attenuated in cells treated with palmitate and aspalathin (p < 0.001) (Fig 7D). Glycolytic reserve was calculated to determine the capability of a cell to respond to an energetic demand, and palmitate-treated cells showed inability to respond to energy demands, while treatment with aspalathin enhanced glycolytic reserve (p < 0.001) (Fig 7E). Insulin stimulation did not further improve the basal effects of aspalathin.

## Discussion

The rapid rise in deaths due to noncommunicable diseases warrants further investigation into novel drugs that can act broadly to alter energy metabolism or influence pathways that contribute to metabolic disease-associated complications. Hepatocytes play a major role in regulating energy metabolism, in addition to their established function in the detoxification of metabolites and synthesis of proteins. Abnormal hepatic energy metabolism has been associated with the development of various metabolic complications, including NAFLD, insulin resistance, and diabetes mellitus [7, 14, 35]. C3A cells are known to exhibit many liver-specific features, including gluconeogenesis and glycogen synthesis in response to insulin stimulation. These cells also express GLUT2 and produce lipid intermediates such as cholesterol [35]. On the other hand, exposure to high palmitate concentrations has been successfully used to set up models of insulin resistance in many cell types, including C3A liver cells [8, 14, 31]. To date, studies reporting on the therapeutic potential of aspalathin against liver associated metabolic complications are lacking. The current study tested the beneficial effects of aspalathin against palmitate-induced hepatic insulin resistance using C3A cells.

Exposing C3A cells to high palmitate concentrations led to a number of metabolic and cellular signaling abnormalities that are related to the development of insulin resistance. Such abnormalities included altered substrate metabolism, mainly characterized by suppressed glucose and FFA uptake, as well as reduced GLUT2 expression, occurring concomitant to altered insulin signaling. Reduced phosphorylation of both PI3K and AKT could explain impaired insulin signaling and development of insulin resistance, as previously demonstrated [8, 13, 31]. Down-regulated expression of CPT1, which is required for mitochondrial FFA uptake, could explain malfunctioning β-oxidation and obstructed energy metabolism. In any case, mitochondria are the main site for lipid oxidation and a connection between reduced FFA uptake, as well as diminished ATP production and compromised respiratory chain function has been previously reported [14, 36]. In agreement, the current study showed that palmitate insult not only impaired substrate metabolism, but also suppressed the glycolytic rate and altered mitochondrial bioenergetics, which could have caused depletion in ATP production. Both glycolysis and mitochondrial respiration reactions are known to be responsible for the major production of ATP in mammals [37]. Therefore, as recently reviewed [18], regulating mitochondrial respiration to increase hepatic energy expenditure, along with improving insulin sensitivity, could be an effective strategy to curb complications associated with the metabolic syndrome, including insulin resistance.

The major findings in this paper suggest that aspalathin can provide a number of beneficial effects that are essential in ameliorating hepatic-induced insulin resistance. Such benefits include tight regulation of hepatic substrate metabolism, which could have led to improved insulin signaling and mitochondrial bioenergetics. As reported by our group and others in different tissue types [8, 27, 28, 31], the modulatory effect of PI3K/AKT as well as AMPK pathways, could be a possible mechanism by which this phytochemical improves insulin signaling and enhance hepatic energy expenditure. Apparently, some of the commonly used diabetic drugs such as insulin and metformin are also known to induce their therapeutic effects through the modulatory effect of PI3K/AKT and AMPK pathways, resulting in improved substrate metabolism and insulin signaling [35, 38]. Similarly, natural products like resveratrol or magnolol can modulate AMPK activity to inhibit lipid-induced toxicity in liver cells [39, 40]. Recent evidence also shows that celastrol, a tripterine isolated from the root extracts of *Tripterygium wilfordii* plant, could ameliorate hepatic insulin resistance by improving insulin signaling and mitochondrial function [41]. Similar to these compounds, Ulicná and colleagues recently showed that rooibos attenuates carbon tetrachloride-induced-induced injury to mitochondrial respiratory function and energy production in rat liver [42]. Whereas, Hattingh and co-workers found that activation of AMPK was necessary for amelioration of ethidium bromide induced mitochondrial dysfunction in 3T3-L1 adipocytes [43]. Thus, we hypothesize that aspalathin, in addition to improving insulin signaling, is likely to target the mitochondrion to increase energy expenditure and reverse insulin resistance. These effects can potentially lead to the amelioration of metabolic syndrome associated complications, including inflammation and oxidative stress. Although not investigated in the current study, existing evidence shows that aspalathin or an aspalathin-enriched enriched rooibos extract can counteract oxidative stress under conditions of metabolic dysregulation in the liver [44–46]. However, this study demonstrated that aspalathin showed limited activity to enhance insulin-sensitizing effects *in vitro*. This result is consistent with our recent findings [29], showing that a rooibos extract containing high levels of aspalathin displayed an ameliorative effect when used in combination with insulin to reverse palmitate-induced hepatic insulin resistance. Nonetheless, additional work, especially an investigation of transcriptional factors involved in mitochondrial bioenergetics, is necessary to support such a hypothesis. This includes mechanistic studies, either making use of genetically mutated rodents or gene-specific silencing *in vitro*, can be essential tools in establishing the involvement of PI3K/AKT or AMPK in the therapeutic effect of aspalathin.

## Conclusion

This study showed that aspalathin can target the liver cells to regulate hepatic cellular metabolism and increase energy expenditure likely by modulating PI3K/AKT and AMPK signaling pathways. However, before proposals presented in this study are accepted, several issues need to be resolved first. This includes confirming these results in an established *in vivo* model of insulin resistance. Such information would not only improve our current understanding on the mechanisms involved in metabolic disease associated liver dysfunction, but would also highlight the potential use of aspalathin as a nutraceutical to protect against metabolic disease-related complications.

## Supporting information

S1 Dataset Original Western blot picture of glucose transporter (GLUT) 2, experiment 1(A), experiment 2(B), experiment 3 (C) and representative cropped blot (D). C3A liver cells were cultured in EMEM supplemented with 8 mM glucose with or without 0.75 mM palmitate for 16 h, and then treated with aspalathin 10 μM for 3 h. Insulin (1 μM) was added during the last 15 min and used as a positive control. Cells were lysed and subjected to Western blot analyses. All 3 independent experiments were analysed and one representative blot was cropped to be included in the article. Results are from three independent experiments. Please note: G-represents “normal control”, GI is “normal control+ insulin”, P is “palmitate control”, PI is “palmitate+insulin”, GRE is “green rooibos extract”, GRE+I is “green rooibos extract + insulin”, ASP is “aspalathin”, and ASP+I is “aspalathin+ insulin”.
(DOCX)

S2 Dataset Original Western blot picture of phosphor-protein kinase B (pAKT) and total-protein kinase B (tAKT), experiment 1 (A, B), experiment 2 (C, D), experiment 3 (E, F) and cropped blot (G). C3A liver cells were cultured in EMEM supplemented with 8 mM glucose with or without 0.75 mM palmitate (Pal) for 16 h, thereafter treated with aspalathin 10 μM for 3 h. Insulin (1 μM) was added during the last 15 min and used as a positive control. Cells were lysed and subjected to Western blot analyses. After probing with pAKT blots were stripped and probed with tAKT. The % of pAKT/tAKT, was used to estimate the level of AKT (Ser 473). All 3 independent experiments were analysed and one representative blot was cropped to be included in the article. Results are from three independent experiments. Please note: G-represents “normal control”, GI is “normal control+ insulin”, P is “palmitate control”, PI is “palmitate+insulin”, GRE is “green rooibos extract”, GRE+I is “green rooibos extract + insulin”, ASP is “aspalathin”, and ASP+I is “aspalathin+ insulin”.
(DOCX)

S3 Dataset Original Western blot picture for phosphor-phosphoinositide 3-kinase (pPI3K) and total-phosphoinositide 3-kinase (tPI3K), experiment 1 (A, B), experiment 2 (C, D), experiment 3 (E, F) and cropped blot (G). C3A liver cells were cultured in EMEM supplemented with 8 mM glucose with or without 0.75 mM palmitate (Pal) for 16 h, thereafter treated with aspalathin 10 μM for 3 h. Insulin (1 μM) was added during the last 15 min and used as a positive control. Cells were lysed and subjected to Western blot analyses. After probing with pPI3K blots were stripped and probed with tPI3K. The % of pPI3K/tPI3K, was used to estimate the level of PI3K (p85). All 3 independent experiments were analysed and one representative blot was cropped to be included in the article. Results are from three independent experiments. Please note: G-represents “normal control”, GI is “normal control+ insulin”, P is “palmitate control”, PI is “palmitate+insulin”, GRE is “green rooibos extract”, GRE+I is “green rooibos extract + insulin”, ASP is “aspalathin”, and ASP+I is “aspalathin+ insulin”.
(DOCX)

S4 Dataset Original Western blot picture of carnitine palmitoyltransferase 1 (CPT1), experiment 1(A), experiment 2(B), experiment 3 (C) and cropped blot (D). C3A liver cells were cultured in EMEM supplemented with 8 mM glucose with or without 0.75 mM palmitate (Pal) for 16 h, and then treated with aspalathin 10 μM for 3 h. Insulin (1 μM) was added during the last 15 min and used as a positive control. Cells were lysed and subjected to Western blot analyses. All 3 independent experiments were analysed and one representative blot was cropped to be included in the article. Results are from three independent experiments. Please note: G-represents “normal control”, GI is “normal control+ insulin”, P is “palmitate control”, PI is “palmitate+insulin”, GRE is “green rooibos extract”, GRE+I is “green rooibos extract + insulin”, ASP is “aspalathin”, and ASP+I is “aspalathin+ insulin”.
(DOCX)

S5 Dataset Original Western blot picture of phosphor-AMP-activated protein kinase (pAMPK) and total-AMP-activated protein kinase (tAMPK), experiment 1 (A, B), experiment 2 (C, D), experiment 3 (E, F) and cropped blot (G). C3A liver cells were cultured in EMEM supplemented with 8 mM glucose with or without 0.75 mM palmitate (Pal) for 16 h, thereafter treated with aspalathin 10 μM for 3 h. Insulin (1 μM) was added during the last 15 min and used as a positive control. Cells were lysed and subjected to Western blot analyses. After probing with pAMPK blots were stripped and probed with tAMPK. The % of pAMPK/tAMPK, was used to estimate the level of AMPK (Thr172). All 3 independent experiments were analysed and one representative blot was cropped to be included in the article. Results are from three independent experiments. Please note: G-represents “normal control”, GI is “normal control+ insulin”, P is “palmitate control”, PI is “palmitate+insulin”, GRE is “green rooibos extract”, GRE+I is “green rooibos extract + insulin”, ASP is “aspalathin”, and ASP+I is “aspalathin+ insulin”.
(DOCX)

S6 Dataset Raw experimental data. (XLSX)

S1 Table List of antibodies and relevant dilutions used in the current study. (DOCX)

## Abbreviations

AKT: protein kinase B
AMPK: 5ʹ-adenosine monophosphate (AMP)-activated protein kinase
ASP: aspalathin
CPT1: carnitine palmitoyl transferase 1
DPBS: Dulbecco’s phosphate buffered saline
ECAR: extracellular acidification rates
EMEM: Eagle's Minimum Essential Medium
FCCP: carbonyl cyanide-p-trifluoromethoxyphenylhydrazone
FFA: free fatty acid
GLUT: glucose transporter
MTT: 3-(4,5-dimethylthiazol-2-yl)-2,5-diphenyltetrazolium bromide
NAFLD: nonalcoholic fatty liver disease
OCR: oxygen consumption rate
PI3K: phosphatidylinositol-4,5-bisphosphate 3-kinase
