# Supplementary material for: Aspalathin, a natural product with the potential to reverse hepatic insulin resistance by improving energy metabolism and mitochondrial respiration
Source: PLoS One. 2019 May 2;14(5):e0216172. doi: 10.1371/journal.pone.0216172 (PMC6497260; doi:10.1371/journal.pone.0216172)
Supplement: S4 Dataset — Original Western blot picture of carnitine palmitoyltransferase 1 (CPT1), experiment 1(A), experiment 2(B), experiment 3 (C) and cropped blot (D). C3A liver cells were cultured in EMEM supplemented with 8 mM glucose with or without 0.75 mM palmitate (Pal) for 16 h, and then treated with aspalathin 10 μM for 3 h. Insulin (1 μM) was added during the last 15 min and used as a positive control. Cells were lysed and subjected to Western blot analyses. All 3 independent experiments were analysed and one representative blot was cropped to be included in the article. Results are from three independent experiments. Please note: G-represents “normal control”, GI is “normal control+ insulin”, P is “palmitate control”, PI is “palmitate+insulin”, GRE is “green rooibos extract”, GRE+I is “green rooibos extract + insulin”, ASP is “aspalathin”, and ASP+I is “aspalathin+ insulin”. (DOCX) [file pone.0216172.s004.docx]

**Aspalathin, a natural product with the potential to reverse hepatic insulin resistance by improving energy metabolism and mitochondrial respiration**

Sithandiwe E. Mazibuko-Mbeje^1,2^ Phiwayinkosi V. Dludla^1,3^, Rabia Johnson^1,2^, Elizabeth Joubert^4,5^, Johan Louw^2,6^, Khanyisani Ziqubu^2,6^, Luca Tiano^3^, Sonia Silvestri^3^, Patrick Orlando^3^, Andy R. Opoku^6^, Christo J.F. Muller^1,2,6^

^1^Biomedical Research and Innovation Platform, South African Medical Research Council, Tygerberg 7505, South Africa.

^2^Division of Medical Physiology, Faculty of Health Sciences, Stellenbosch University, Tygerberg 7505, South Africa.

^3^Department of Life and Environmental Sciences, Polytechnic University of Marche, Ancona 60121, Italy.

^4^Plant Bioactives Group, Post-Harvest and Agro-Processing Technologies, Agricultural Research Council, Infruitec-Nietvoorbij, Stellenbosch 7599, South Africa.

^5^Department of Food Science, Stellenbosch University, Stellenbosch 7599, South Africa.

^6^Department of Biochemistry and Microbiology, University of Zululand, KwaDlangezwa 3886, South Africa.

Corresponding author:

Sithandiwe E. Mazibuko-Mbeje, Biomedical Research and Innovation Platform, South African Medical Research Council, Tygerberg 7505, South Africa. Email: sithandiwe.mazibuko@mrc.ac.za. Tel.: +2721 938 0341.

**Material and methods**

**Western blot analysis**

For Western blot analysis, membranes were probed overnight at 4°C with the relevant primary antibodies glucose transporter (GLUT)2, protein kinase B (AKT, p-AKT (Ser 473), 5' AMP-activated protein kinase (AMPK, p-AMPK (Thr172), carnitine palmitoyltransferase 1 (CPT1) and phosphoinositide 3-kinase (PI3K, p-PI3K (p85)) , as well as horseradish peroxidase (HRP) conjugated secondary antibody (β-actin) applied for 1.5h the following day. Chemiluminenscence using a Chemidoc-XRS imager and Quantity One 1-D software (Biorad Laboratories, Hercules, CA, USA) were used detect and quantify proteins. β-actin was used as the reference control.Three independet experimnents were conducted and one blot was selected as a representative. The selected represenative image was croped and image was used to quantify molecular weight size. In some of the the pictures out of 8 lanes loaded only 6 were used as the other treatment [gree rooibos extract (GRE) and green rooibos extract + insulin (GRE+I)] is not discussed in the current article. Molecular weight and band analysis was quantified using image J software.

**Carnitine palmitoyltransferase 1 (CPT1) original Western blots**

Three independent experiments were conducted, in all figures GRE and GRE+I were run on the same blot, however GRE and GRE+I are not discussed in this paper. Similar tread was attained in all blots (Fig. 4A, B and C). Experiment one (Fig.4C) was cropped and used as a representative ( Fig.4D).

A B

Marker kDa G GI PAL PAL+I GRE GRE+I ASP ASP+I Marker kDa

G GI PAL PAL+I GRE GRE+I ASP ASP+I Marker kDa


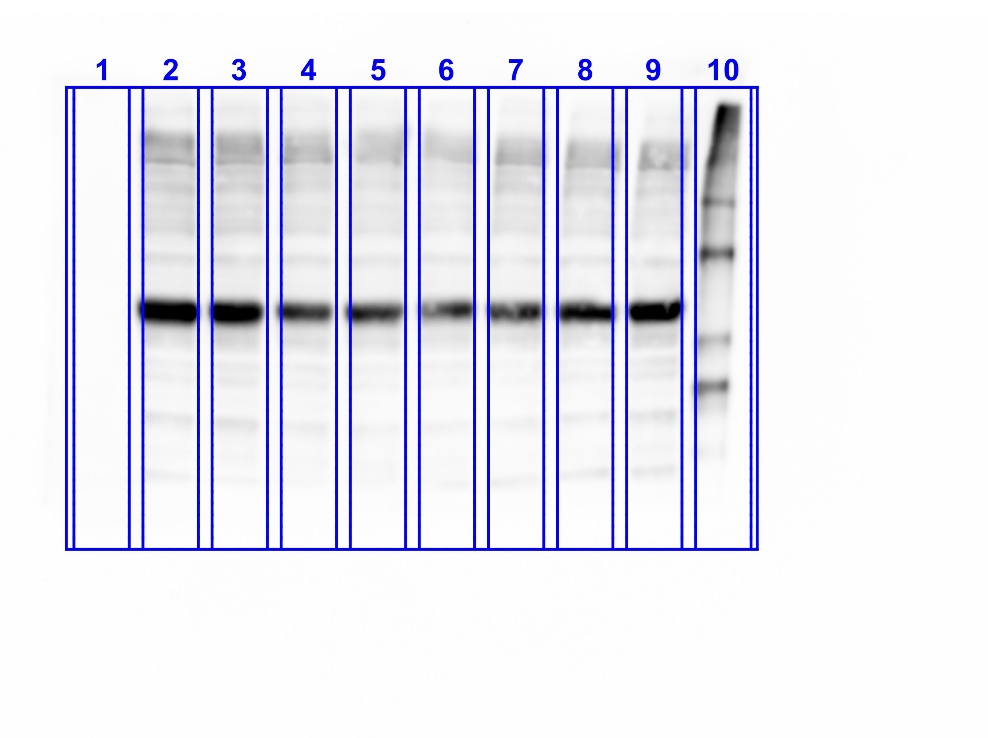

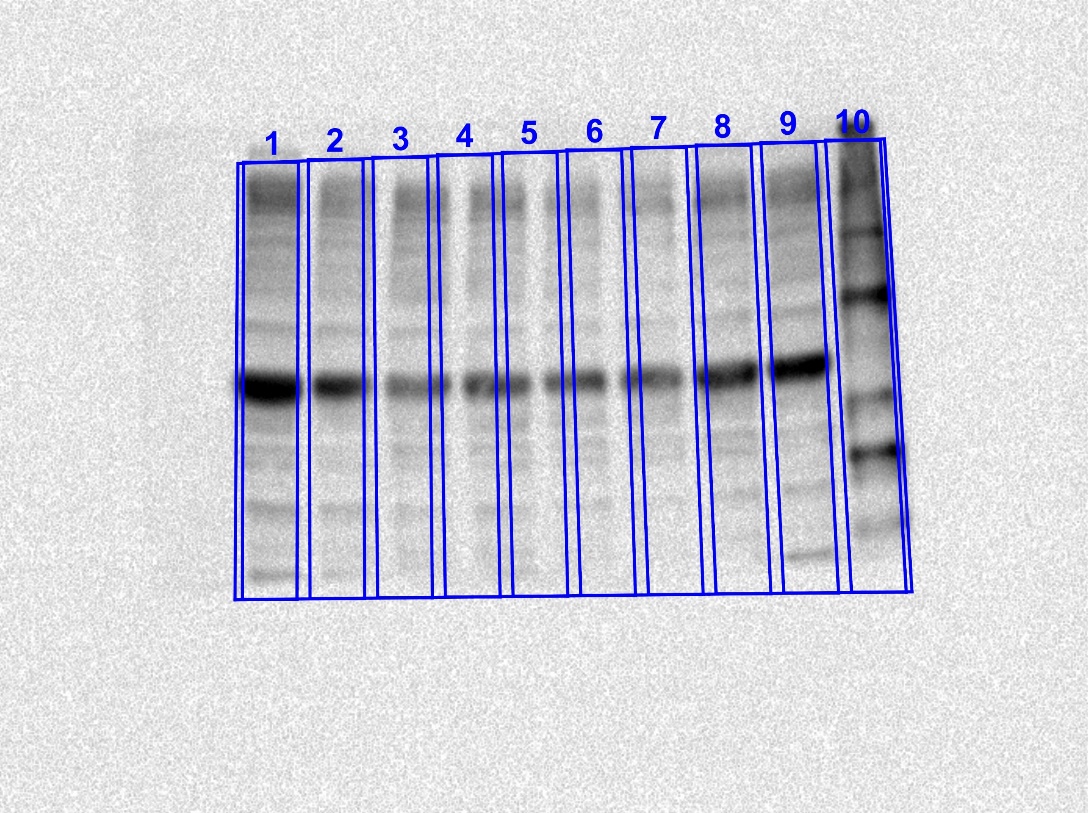


C

~88 CPT1

~88 CPT1

C D


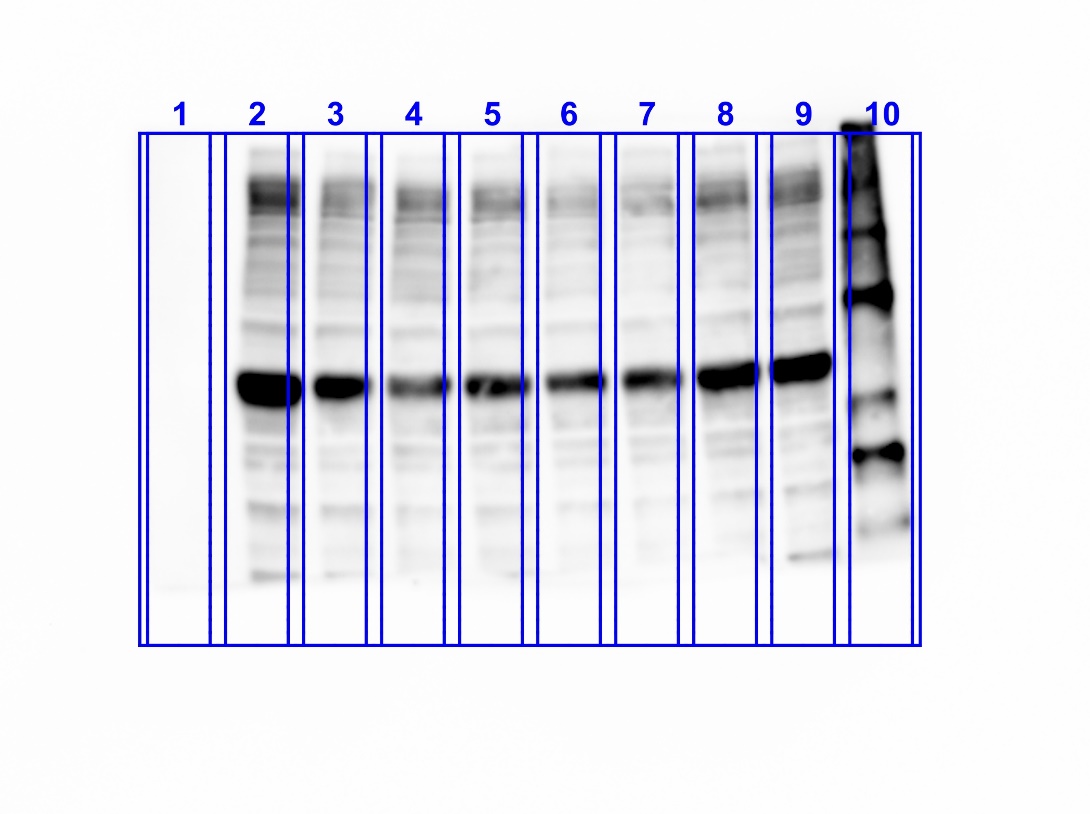


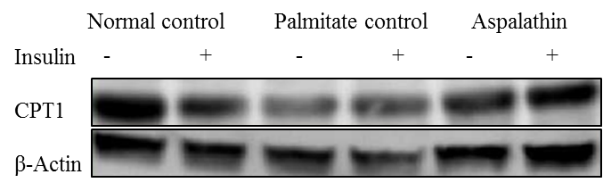


G GI P PI ASP ASP+I

Marker kDa G GI PAL PAL+I GRE GRE+I ASP ASP+I Marker kDa

~88 CPT1

**S4 Data set.**
