# Supplementary material for: Aspalathin, a natural product with the potential to reverse hepatic insulin resistance by improving energy metabolism and mitochondrial respiration
Source: PLoS One. 2019 May 2;14(5):e0216172. doi: 10.1371/journal.pone.0216172 (PMC6497260; doi:10.1371/journal.pone.0216172)
Supplement: S1 Table — (DOCX) [file pone.0216172.s007.docx]

**Aspalathin, a natural product with the potential to reverse hepatic insulin resistance by improving energy metabolism and mitochondrial respiration**

Sithandiwe E. Mazibuko-Mbeje^1,2^ Phiwayinkosi V. Dludla^1,3^, Rabia Johnson^1,2^, Elizabeth Joubert^4,5^, Johan Louw^2,6^, Khanyisani Ziqubu^2,6^, Luca Tiano^3^, Sonia Silvestri^3^, Patrick Orlando^3^, Andy R. Opoku^6^, Christo J.F. Muller^1,2,6^

^1^Biomedical Research and Innovation Platform, South African Medical Research Council, Tygerberg 7505, South Africa.

^2^Division of Medical Physiology, Faculty of Health Sciences, Stellenbosch University, Tygerberg 7505, South Africa.

^3^Department of Life and Environmental Sciences, Polytechnic University of Marche, Ancona 60121, Italy.

^4^Plant Bioactives Group, Post-Harvest and Agro-Processing Technologies, Agricultural Research Council, Infruitec-Nietvoorbij, Stellenbosch 7599, South Africa.

^5^Department of Food Science, Stellenbosch University, Stellenbosch 7599, South Africa.

^6^Department of Biochemistry and Microbiology, University of Zululand, KwaDlangezwa 3886, South Africa.

Corresponding author:

Sithandiwe E. Mazibuko-Mbeje, Biomedical Research and Innovation Platform, South African Medical Research Council, Tygerberg 7505, South Africa. Email: sithandiwe.mazibuko@mrc.ac.za. Tel.: +2721 938 0341.

**Material and methods**

S1 Table. List of antibodies and relevant dilutions used in the current study.

| **Antibody** | **Supplier** | **Catalougue number** | **Dilution** |
| --- | --- | --- | --- |
| AKT | Cell signalling | 9272 | 1:1000 |
| p-AKT (Ser473) | Cell signalling | 9271 | 1:1000 |
| AMPK | Cell signalling | 2532 | 1:800 |
| p-AMPK (Thr172) | Cell signalling | 2531 | 1:800 |
| GLUT2 | Abcam | ab54460 | 1:500 |
| PI3K | Cell signalling | 4292 | 1:800 |
| p-PI3K (p85) | Cell signalling | 5228 | 1:800 |
| CPT1 | Abcam | ab53532 | 1:1000 |
| β-actin | Santa cruz | sc-47778 | 1:200 |
| Donkey-anti mouse | Santa cruz | IgG-HRP: sc-2314 | 1:4000 |
| Donkey-anti rabbit | Santa cruz | IgG-TR: sc-2784 | 1:4000 |
